# A Nonsense Variant in the *ST14* Gene in Akhal-Teke Horses with Naked Foal Syndrome

**DOI:** 10.1534/g3.117.039511

**Published:** 2017-02-22

**Authors:** Anina Bauer, Theresa Hiemesch, Vidhya Jagannathan, Markus Neuditschko, Iris Bachmann, Stefan Rieder, Sofia Mikko, M. Cecilia Penedo, Nadja Tarasova, Martina Vitková, Nicolò Sirtori, Paola Roccabianca, Tosso Leeb, Monika M. Welle

**Affiliations:** * Institute of Genetics, Vetsuisse Faculty, University of Bern, 3001, Switzerland; † DermFocus, University of Bern, 3001, Switzerland; ‡ Swiss Competence Center of Animal Breeding and Genetics, Bern University of Applied Sciences HAFL & Agroscope, University of Bern, 3001, Switzerland; § Institute of Animal Breeding and Genetics, University of Göttingen, 37075, Germany; ** Agroscope, Swiss National Stud Farm, 1580 Avenches, Switzerland; †† Department of Animal Breeding and Genetics, Swedish University of Agricultural Sciences, 75007 Uppsala, Sweden; ‡‡ Veterinary Genetics Laboratory, School of Veterinary Medicine, University of California, Davis, California 95616; §§ Russian Akhal-Teke Association, 115470 Moscow, Russia; *** International Akhal-Teke Association, 115470 Moscow, Russia; ††† Equine Veterinary Practice, 91601 Stará Turá, Slovakia; ‡‡‡ Equine Veterinary Practice, 29010 Agazzano, Italy; §§§ Department of Veterinary Medicine, University of Milan, 20133, Italy; **** Institute of Animal Pathology, Vetsuisse Faculty, University of Bern, 3001, Switzerland

**Keywords:** *Equus caballus*, dermatology, skin, hair, genodermatosis, whole genome sequencing

## Abstract

Naked foal syndrome (NFS) is a genodermatosis in the Akhal-Teke horse breed. We provide the first scientific description of this phenotype. Affected horses have almost no hair and show a mild ichthyosis. So far, all known NFS affected horses died between a few weeks and 3 yr of age. It is not clear whether a specific pathology caused the premature deaths. NFS is inherited as a monogenic autosomal recessive trait. We mapped the disease causing genetic variant to two segments on chromosomes 7 and 27 in the equine genome. Whole genome sequencing of two affected horses, two obligate carriers, and 75 control horses from other breeds revealed a single nonsynonymous genetic variant on the chromosome 7 segment that was perfectly associated with NFS. The affected horses were homozygous for *ST14*:c.388G>T, a nonsense variant that truncates >80% of the open reading frame of the *ST14* gene (p.Glu130*). The variant leads to partial nonsense-mediated decay of the mutant transcript. Genetic variants in the *ST14* gene are responsible for autosomal recessive congenital ichthyosis 11 in humans. Thus, the identified equine *ST14*:c.388G>T variant is an excellent candidate causative variant for NFS, and the affected horses represent a large animal model for a known human genodermatosis. Our findings will enable genetic testing to avoid the nonintentional breeding of NFS-affected foals.

Spontaneous mutants in domestic animals represent valuable animal models for genetic research. Several such mutants, with either missing or altered hair characteristics, have contributed to our knowledge on hair follicle development and hair growth. These include hairless dog breeds, such as the Chinese Crested, Mexican Hairless, and Peruvian Hairless dogs, which show an ectodermal dysplasia phenotype characterized by missing hair and altered dentition due to a genetic variant in the *FOXI3* gene (Drögemüller *et al.* 2008). A genetic variant in the *KRT71* gene is responsible for the hairless Sphynx cats, and other variants in *KRT71* cause curly hair in dogs and cats (Cadieu *et al.* 2009; Gandolfi *et al.* 2010, 2013). In contrast to these breed-defining characters, several hair-related traits with very severe effects on other organ systems are actively selected against. An example for this latter group is the “hairlessness with short life expectancy” in *FOXN1* mutant cats that involves a severe immunodeficiency, in addition to the missing hair, and thus resembles *Foxn1* mutant *nude* mice and rats (Nehls *et al.* 1994; Abitbol *et al.* 2015).

The Akhal-Teke horse breed has its origin in central Asia and is known for high endurance and the characteristic metallic shine of its hair (Leisson *et al.* 2011). The Akhal-Teke studbook is closed since 1932. Due to the limited gene pool and the practice of line breeding, there is a high degree of inbreeding in the Akhal-Teke breed, which favors the appearance of recessive genetic disorders (Leroy and Baumung 2011). The naked foal syndrome (NFS) is a genetic disorder in the Akhal-Teke breed that is known among breeders (Kuznetcova *et al.* 2006; Kuznetcova 2006). However, according to our knowledge, NFS has never been described in the scientific literature. Horses with this disorder are born hairless, and often die within days to months after birth. However, the reason for these early deaths is not known, and some hairless foals have survived up to 2.5 yr. The first records of hairless Akhal-Teke foals date back to 1938, and since then the number of such foals has increased steadily. Many horses with NFS might have been registered as stillborn or weak born, or not been reported at all (Kuznetcova *et al.* 2006).

The aim of the present study was to identify the causative genetic variant for NFS, and to develop a genetic test enabling the identification of carriers. As this phenotype has never been fully described in the scientific literature, we also present a preliminary qualitative characterization of the NFS phenotype.

## Materials and Methods

### Ethics statement

Animal work consisted of collecting blood samples and skin biopsies of privately owned horses with owners’ consent, in accordance with the relevant local guidelines (permits no. BE31/13 and BE75/16).

### Samples and genotyping

The study included 206 Akhal-Teke horses, including five NFS affected horses and 10 obligate carriers (= owner-reported parents of NFS affected animals, Supplemental Material, Table S1). Genomic DNA was isolated from EDTA blood, or hair samples, or, in the case of one deceased affected foal, from a hoof that had been archived. In addition to the Akhal-Teke horses, we used genomic DNA samples from 400 control horses of different breeds that had been collected in the course of other projects and were stored in our biobank. We additionally collected blood from a carrier in a PAXgene blood RNA tube to enable isolation of RNA. Genotyping of four affected horses and four obligate carriers was performed by GeneSeek/NeoGene on the Affymetrix equine 670 k SNP array containing 670,796 evenly distributed markers.

### Skin biopsy sampling and histopathology

Three 8-mm punch biopsies of an NFS affected, an obligate carrier, and a control Akhal-Teke horse were collected and fixed in 4% buffered formalin. Wedge biopsies were taken from a second NFS affected Akhal-Teke foal after euthanasia. We processed the tissue, and stained the skin sections with hematoxylin and eosin (H&E) prior to histopathological examination by a board certified veterinary pathologist (M.W.).

### Linkage analysis

We used genotype data from an Akhal-Teke family consisting of three NFS affected half-siblings, and their four parents, to perform a parametric linkage analysis (Figure S1). For all horses, the call rate was >95%. Using PLINK v1.07 (Purcell *et al.* 2007), we removed markers that were noninformative, located on the sex chromosomes, missing in any of the seven horses, had Mendel errors, or a minor allele frequency <0.2. The final pruned dataset contained 210,556 markers. To analyze the data for parametric linkage, we applied an autosomal recessive inheritance model with full penetrance and a disease allele frequency of 0.7, and the Merlin software (Abecasis *et al.* 2002).

### Homozygosity mapping

Affymetrix equine 670 k SNP genotypes were available for four affected horses. One of these cases was excluded from further analysis because of a low call rate of <95%. We further excluded markers that were missing in one of the three remaining cases, markers on the sex chromosomes, and markers with Mendel errors in the family (see above). Using the --homozyg and --homozyg-group options in PLINK, we searched for extended regions of homozygosity >1 Mb. We visually inspected the raw genotypes to compile the exact boundaries of the homozygous intervals in an Excel file.

### Whole genome sequencing of two affected horses and two carriers

We prepared illumina PCR-free TruSeq fragment libraries with an insert size of ∼350 bp from two cases (AKT001 and AKT006) and two carriers (AKT003 and AKT004) and collected 19× to 33× coverage 2 × 150 bp paired-end data on an Illumina HiSeq 3000 instrument. Read mapping and variant calling was done as described previously (Murgiano *et al.* 2016).

### Sanger sequencing

We used Sanger sequencing to genotype all available Akhal-Teke horses and 400 control horses of different breeds for the *ST14*:c.388G>T variant. A genomic fragment containing the variant position was PCR-amplified from genomic DNA with the primers F, CTGAGAGCAGAGGGTCAAGG, and R, GTGCACTGGCTCTGTGACTG, using AmpliTaqGold360Mastermix (Life Technologies). We directly sequenced the PCR products using the PCR primers on an ABI 3730 capillary sequencer (Life Technologies) after treatment with exonuclease I and shrimp alkaline phosphatase. Sequence data were analyzed using Sequencher 5.1 (GeneCodes).

### RNA isolation and RT-PCR

RNA was isolated from the PAXgene blood of a carrier using the PAXgene Blood RNA kit (PreAnalytiX, Qiagen), and a reverse transcription PCR was performed using Superscript IV reverse transcriptase (Life Technologies). Subsequently, a cDNA fragment was PCR amplified using the primers F, CTGGCCAATAAGGTGAAGGA and R, CCTGTGTGGTCTGTGCTGTT with AmpliTaqGold360Mastermix (Life Technologies). The RT-PCR product and the genomic PCR product described above were sequenced using the same primer SEQ, AAAGCCACCACCGAGGTC, for the sequencing reaction.

### Reference genome assembly and gene annotation

The horse EquCab 2.0 reference genome assembly was used for all analyses. Numbering within the equine *ST14* gene corresponds to the NCBI accessions XM_005611718.2 (mRNA) and XP_005611775.1 (protein). Numbering within the human gene and protein corresponds to the accessions NM_021978.3 and NP_068813.1, respectively.

### Data availability

Figure S1 illustrates the pedigrees of the NFS-affected horses. Table S1 gives additional information on the five NFS-affected and 10 obligate carrier horses used in this study. Genotypes for the *ST14*:c.388G>T variant from 154 Akhal-Teke horses and 400 control horses are listed in Table S2. Table S3 lists genome regions ≥1 Mb that showed positive LOD scores in the linkage analysis. In Table S4, homozygous genome regions ≥1 Mb with shared alleles among three NFS affected Akhal-Teke horses are given. The genome sequencing data of the Akhal-Teke horses were deposited in the European Nucleotide Archive, under accession PRJEB14779. Accession numbers of sequencing data from control horses can be found in Table S5. 

## Results

### Clinical and pathological phenotype

#### Case 1:

**Figure 1:**
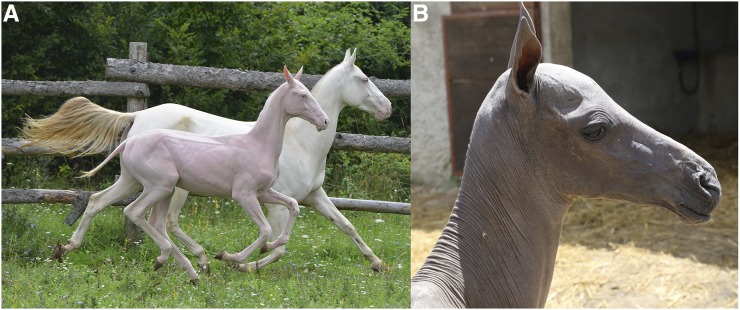
NFS phenotype. (A) An NFS affected colt (case 1) in front of its nonaffected mother. The affected colt has almost no hair. Both horses are of cremello coat color (*SLC45A2*:c.457G>A), which leads to a very strong dilution of the pigmentation but is unrelated to the NFS phenotype. (B) NFS affected filly (case 2).

We examined two Akhal-Teke horses with NFS. Case 1 was a male cremello foal born in March 2014 (Figure 1A). This horse was still alive at the time of manuscript revision (January 2017, 2 yr 10 months). Compared to nonaffected horses of the same age, and raised at the same stud, it had a growth delay and was small for its age. The horse was alopecic, with only sparse and thin body hairs. In both fore and hind limbs, the proximal parts were completely alopecic, while the hair density increased toward the distal ends of the limbs. Mane and hairs at the tail were sparse or absent. Whiskers were present but sparse, curly, and abnormally short. Eyelashes were missing. The skin was dry and scaly (xerosis cutis) in some body parts. Furthermore, a persisting increase in tear flow (epiphora) was reported by the owner. Multifocal scars and erosive lesions were present, possibly due to the missing physical protection of a normal hair coat. We did not observe any abnormalities in the teeth or hooves.

#### Case 2:

**Figure 2:**
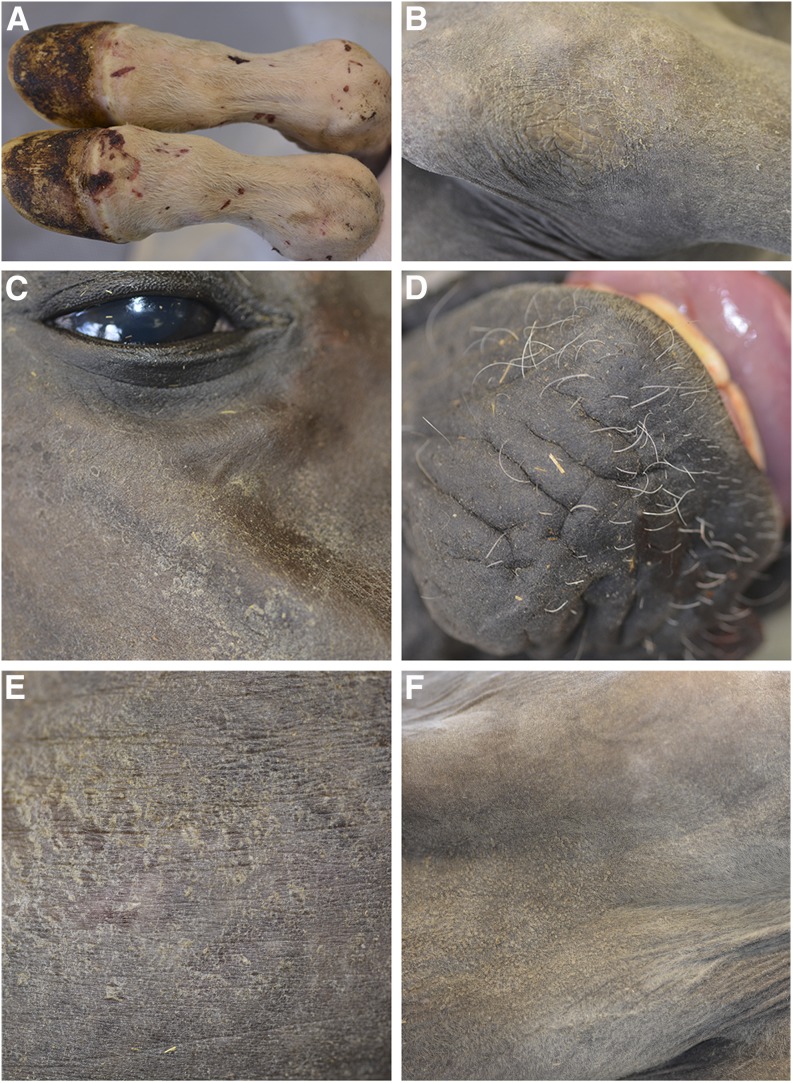
Clinical findings in a 3-wk-old foal with NFS (case 2). (A) Distal fore limbs showed the presence of sparse hairs and multiple abrasive lesions. (B) Dry and scaly skin on the proximal forelimb (xerosis cutis). (C) Scaly alopecic area on the head and missing eyelashes. (D) Sparse, curly, and abnormally short whiskers on the muzzle. (E) Dry and scaly skin on the flank. (F) Hyperkeratosis in the pectoral region.

Case 2 was a female, born in June 2016 (Figure 1B). The skin and hair phenotype closely resembled that of case 1 (Figure 2). Hooves and teeth were also normal. This foal had to be killed at 21 d of age due to a spontaneous leg fracture. A full necropsy on the killed foal was performed. Gross findings revealed a mild internal hydrocephalus, a heart defect (dysplasia of the tricuspidal valve), and severely altered lymphoid organs. The histological findings in the lymphoid organs were consistent with a defect in primary immune organ development, and the specific immune response. Specifically, the thymus lacked cortico-medullary organization, and was characterized by absence of Hassal corpuscles. Abnormal or absent T cell zones were noted in the spleen and the peripheral lymph nodes.

### Histopathologic findings

**Figure 3:**
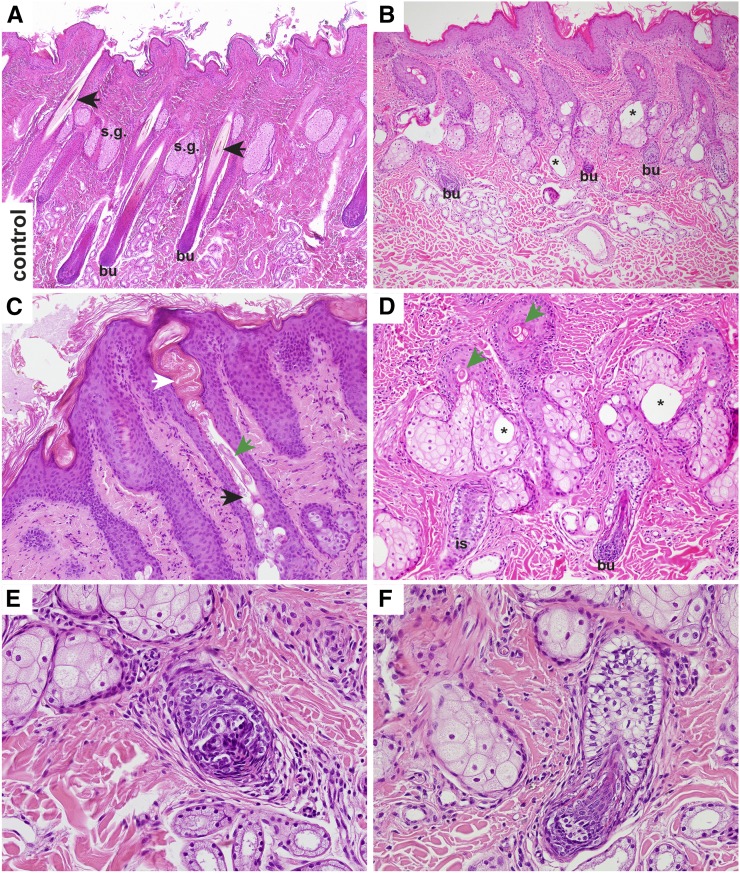
Histopathology of a control and an NFS affected horse (H&E staining). (A) In the control horse, long anagen hair follicles extended into the subcutis, and the bulb (bu) reached far below the level of sebaceous glands (s.g.). The hair follicles contained approximately equally sized, and normally differentiated, hair shafts (arrows). Only little keratin and no sebum were visible in the infundibula. Sebaceous glands had no vacuoles. (B) In the affected horse, the shortened anagen follicles ended at the level of, or just below, the sebaceous glands. There were large empty vacuoles within the sebaceous glands (asterisks). (C) Higher magnification of an infundibulum of an affected horse. The infundibulum contained an increased amount of keratin (white arrow) and sebum (black arrow). The hair shaft present was thin, and had an abnormal structure (green arrow). (D) Higher magnification of the proximal portion of the dysplastic hair follicles. The hair shafts were very thin (green arrows), and the isthmus (is) and bulbar region (bu) appeared only as remnants. Note also the large empty vacuoles within the sebaceous glands. (E) Dysplastic hair bulb in close vicinity to the sebaceous glands of an affected horse. The matrical cells were irregularly arranged, not equal in size, and sometimes vacuolated. (F) Another example of a very small dysplastic bulb in comparison to the adjacent lower isthmus. Note the larger vacuoles within some matrical cells.

We performed a histological examination of skin sections from two NFS-affected horses, an obligate carrier, and a control horse. Histologic examination of skin from the carrier, and the unrelated control, were not noticeably different. Both affected horses revealed severely shortened anagen follicles, in which only remnants of the isthmic portion were present, and the hair bulbs of the majority of the follicles were located at the level of, or only slightly below, the sebaceous glands. The infundibula of the hair follicles were often distorted and filled with excessive infundibular keratin, and, especially at the level of the entrance of the sebaceous duct, with abundant sebum. Furthermore, the follicular lumen was often distended at the level of the sebaceous duct entrance. The epithelium of the infundibula and the rudimentary isthmi were mild to moderately hyperplastic and irregular. The sebaceous glands presented with large empty vacuoles. The hair shafts, if present, were very thin and lacking the normal structure. The dysplastic hair bulbs differed in size, and the matrical cells of the bulbs were arranged irregularly. Sometimes larger vacuoles were present. The epidermis was moderately hyperplastic, and covered with moderate orthokeratotic laminar to basket weave keratin. In addition, there was a mild perivascular to interstitial lymphocytic infiltrate in the dermis. The histological findings in the hair follicles were compatible with a follicular dysplasia (Figure 3).

### Mapping of candidate regions

As the breeders’ records indicated a monogenic autosomal recessive mode of inheritance, we used a combined linkage and homozygosity mapping approach to establish the position of the causative variant in the genome (Figure S1). SNP microarray genotypes from three NFS affected half-siblings and their four parents were determined. We performed a parametric linkage analysis assuming a fully penetrant autosomal recessive inheritance model, and obtained 50 linked genomic regions of >1 Mb with a positive LOD score on 26 different chromosomes (Table S3).

**Figure 4:**
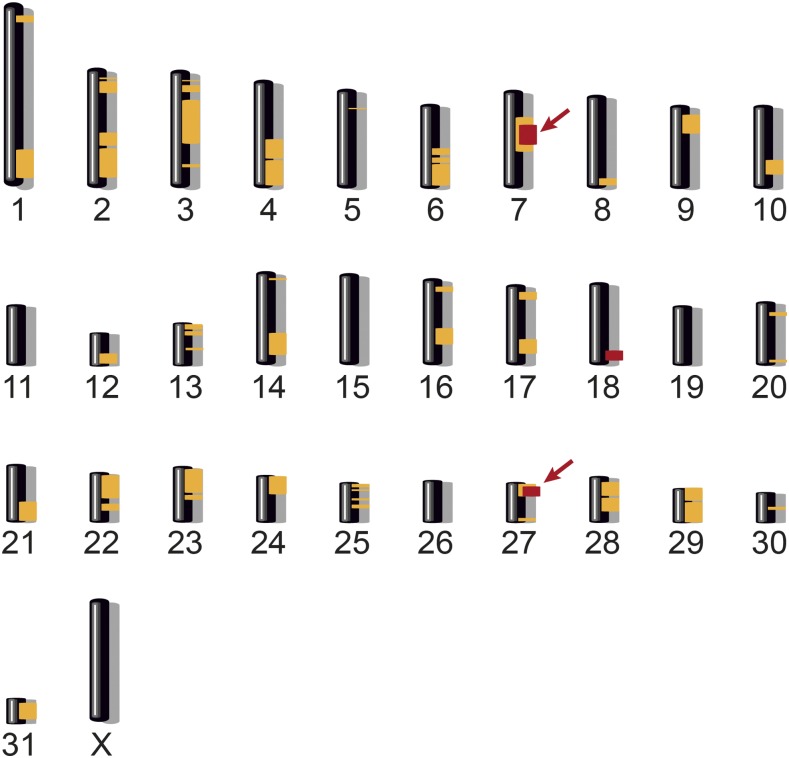
Combined linkage and homozygosity mapping. The 50 linked chromosomal segments >1 Mb are shown in yellow. Homozygous intervals >1 Mb located on chromosomes 7, 18, and 27 are indicated in red. Intersecting the regions obtained by the two methods resulted in critical intervals on chromosomes 7 and 27 (arrows).

We then searched for extended regions of homozygosity in the same three affected horses that had been used for the linkage analysis. Six intervals on chromosomes 7, 18, and 27 fulfilled our homozygosity search criteria (Figure 4 and Table S4). Intersecting the intervals obtained by linkage analysis and homozygosity mapping resulted in four critical intervals located on chromosome 7 and one interval on chromosome 27. Taken together, the four chromosome 7 intervals spanned >15 Mb, and were interrupted only by three small nonhomozygous segments of <1.5 Mb. We therefore conservatively considered the four segments as one whole interval on chromosome 7 for all further analyses. This ∼17 Mb interval spanned from position 36,013,146 to position 53,111,271 on chromosome 7. The single 1.2 Mb interval on chromosome 27 spanned from 10,880,351 to 12,070,934.

### Whole genome sequencing and identification of the causative variant

To further characterize the identified genomic regions on chromosome 7 and 27, we sequenced the genomes of two affected horses and two obligate carriers at ∼20× to 33× coverage. For each sample, we called single nucleotide variants and small indels with respect to the equine reference genome assembly. Since the disorder is only known in the Akhal-Teke breed, we hypothesized that the causative variant should be completely absent in horses of other breeds. We therefore filtered for variants that were present in homozygous state in the affected horses, in heterozygous state in the carriers, and absent or missing in 75 control horses of different breeds.

In the interval on chromosome 27, none of the variants fulfilled our search criteria. In the 17 Mb region on chromosome 7, we found four variants that showed perfect cosegregation with NFS (Table 1). Among these variants, the only nonsynonymous variant was a nonsense variant in the *ST14* gene (c.388G>T) introducing a premature stop codon into exon 4. *ST14* encodes “suppression of tumorigenicity 14,” a type II serine protease that is highly expressed in the epidermis, and was reported to be essential for epidermal barrier function, and development of hair follicles (List *et al.* 2002).

**Table 1:** Variants detected by whole genome resequencing of two cases, two obligate carriers, and 75 controls

Filtering Step	Number of Variants
Variants with genotypes 1/1 in cases and 0/1 in carriers	53,683
NFS-associated variants in whole genome*^a^*	351
NFS-associated variants in critical interval on chr. 27*^a^*	0
NFS-associated variants in critical interval on chr. 7*^a^*	4
NFS-associated nonsynonymous variants in critical intervals*^a^*	1

a “NFS-associated” indicates variants that were homozygous for the alternate allele in the two cases, heterozygous in the two carriers, and homozygous reference (or missing) in the 75 control horses.

We confirmed the presence of the *ST14*:c.388G>T variant by Sanger sequencing on the genomic and transcript level. In addition, we genotyped all available samples from Akhal-Teke horses, as well as 400 horses from various breeds for this variant. As expected, the variant was absent in all tested horses outside of the Akhal-Teke breed. It was homozygous in the five available affected horses, and heterozygous in 10 obligate carriers (Table 2).

**Table 2:** Association of the *ST14*:c.388G>T variant with NFS

Genotype	G/G	G/T	T/T
Affected horses (*N* = 5)	0	0	5
Obligate carriers (*N* = 10)	0	10	0
Remaining Akhal-Teke horses (*N* = 191)	165	26	0
Control horses from other breeds (*N* = 400)	400	0	0

**Figure 5:**
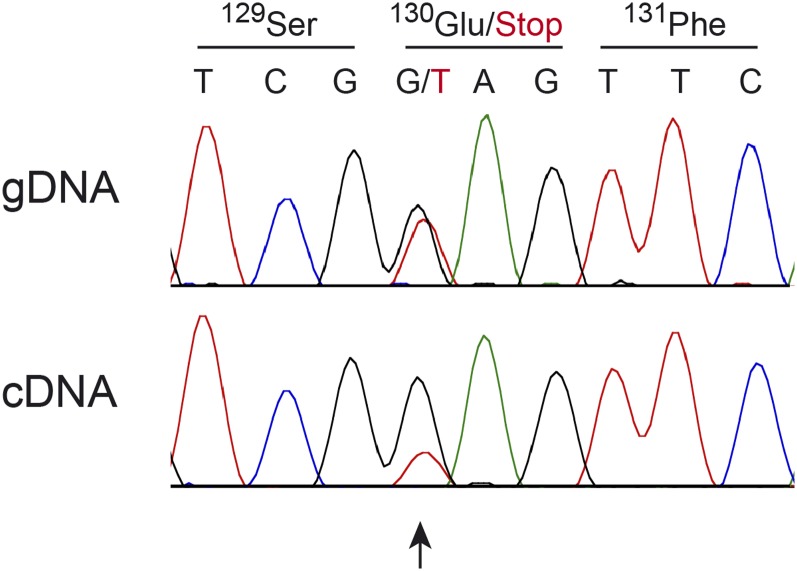
Nonsense-mediated decay of mRNA containing the *ST14*:c.388G>T variant. A fragment containing the variant was amplified from genomic DNA (gDNA), or cDNA of a heterozygous carrier animal and analyzed by Sanger sequencing. The relative peak heights at the heterozygous position differ between sequences amplified from gDNA or cDNA. While the genomic sequence shows the expected 1:1 ratio between the alleles, the mutant allele is underrepresented at the cDNA (transcript) level.

The *ST14*:c.388G>T variant introduces a premature stop codon predicted to truncate >80% of the open reading frame (p.Glu130*). We hypothesized that mRNA carrying this variant is likely to be degraded by nonsense-mediated decay. To test this hypothesis, we isolated RNA from peripheral blood mononuclear cells from an obligate carrier. Sanger sequencing of a genomic DNA fragment, and the corresponding cDNA fragment of this heterozygous animal, indicated partial nonsense-mediated decay of the mutant transcript (Figure 5).

## Discussion

In this study, we used a combined positional mapping and whole genome sequencing approach to unravel the genetic background of NFS in the Akhal-Teke breed. We identified two critical intervals on chromosomes 7 and 27. The *ST14*:c.388G>T nonsense variant was the only nonsynonymous trait-associated variant in the critical intervals. It was perfectly associated with NFS in all tested Akhal-Teke horses, and completely absent from horses of other breeds. Taken together, the genetic association, the fact that *ST14*:c.388G>T is a nonsense variant, and the knowledge on the *ST14* gene from other species, strongly suggest that this is indeed the causative variant for NFS in Akhal-Teke horses.

The suppressor of tumorigenicity 14 gene (*ST14*) encodes a type II serine protease that was also termed matriptase. It was first described in the context of cancer, but later studied as a protease involved in epithelial, and, in particular, epidermal development (Shi *et al.* 1993; Zhang *et al.* 1998; Miller and List 2013). In *St14* deficient knockout mice, all pups homozygous for the null allele died within 48 hr of birth (List *et al.* 2002). They showed malformed epidermal surfaces with a dry, red, wrinkled, and shiny appearance, and had a lower body weight and smaller size than heterozygous or homozygous wildtype littermates. Abnormalities in stratum corneum development, and loss of both inward and outward barrier functions, were reported. As a consequence, the rate of epithelial fluid loss was significantly accelerated in *St14* deficient mice, resulting in rapid postnatal death. Histological examination showed that vesicular bodies, the major source of intercorneocyte lipids, were not present in transitional cells, which could explain the impaired barrier function. Furthermore, vibrissal hairs were absent in *St14* knockout pups, hair follicles were hypomorphic, and the hair shafts were curved and ingrown, possibly due to the absence of the hair canal (List *et al.* 2002).

In humans, *ST14* variants may cause autosomal recessive congenital ichthyosis 11 (ARCI11, OMIM #602400), which can be further subclassified into autosomal recessive ichthyosis with hypotrichosis (ARIH) and ichthyosis, follicular atrophoderma, hypotrichosis, and hypohidrosis (IFAH). In three members of a consanguineous Israeli-Arab family, ARIH was reported to be caused by the p.Gly827Arg missense variant in the *ST14* gene. The patients showed diffuse and generalized hypotrichosis, diffuse scaling and curly, fragile, sparse slowly growing hair, photophobia, and mild abnormalities of the teeth. Similar to horses with NFS, the hair shafts of the human patients were irregular. Further examination of skin samples of a patient revealed that intact corneodesmosomes were present in the upper layers of the stratum corneum, which indicated an impaired degradation of corneodesmosomes, and an involvement of ST14 in desquamation (Basel-Vanagaite *et al.* 2007). A female patient with ARIH caused by the variant c.3G>A, which shifts the translation initiation, did not show any tooth abnormalities, but sparse eyebrows and eyelashes (Avrahami *et al.* 2008). While the ARIH variants were all *ST14* missense variants, one of the IFAH patients had a splice site variant (c.2269+1G>A). In another IFAH patient, a frame shift deletion (c.2034delG) was described. Both variants were predicted to result in premature stop codons. Functional analyses on keratinocytes of the patient with the deletion variant revealed that ST14 was completely missing, profilaggrin processing was altered, and prostasin, a GPI-anchored serine protease with a role in the same proteolytic cascade as ST14, was found only in its inactive form (Alef *et al.* 2009).

Reduced proteolytic activation of prostasin, and reduced processing of profilaggrin were also found in *St14* hypomorphic mice that developed a phenotype comparable to ARIH (List *et al.* 2007). A study on *St14* deficient mice further demonstrated that filaggrin monomers, and the filaggrin S-100 protein that promotes terminal differentiation of keratinocytes were missing, indicating a specific role for ST14 in these processing steps, and in terminal epidermal differentiation (List *et al.* 2003).

Taken together, the *ST14* gene has essential roles in the interfollicular epidermis by contributing to epidermal barrier formation as well as for hair follicle development. The observed phenotype in NFS-affected horses resembles the group of heterogeneous phenotypes caused by *ST14* variants in humans and mice. However, in horses with NFS, the degree of alopecia is more severe than in human patients, whereas the ichthyosis is less pronounced, and suggested mainly by the clinical picture. It is not fully clear why NFS affected foals have such a short life expectancy. While none of the known NFS foals so far became older than 3 yr, there also was huge variability, with some foals dying at a few weeks of age, and others surviving at least for >2 yr. Given the variability in life span, we consider the hydrocephalus, as well as the heart and immune system changes, in our case 2 as coincidental findings unrelated to NFS, but this should be reinvestigated if further NFS-affected foals become available for pathologic examination. Further research will be necessary to characterize the pathologies leading to premature death in these horses.

In conclusion, we provide an initial scientific description of the phenotype of NFS, a trait that has been segregating in the Akhal-Teke breed for >75 yr. Furthermore, we identified the *ST14*:c.388G>T nonsense variant as most likely underlying genetic defect. These findings thus provide a potential large animal model for related human diseases, and allow genetic testing to avoid the nonintentional breeding of NFS-affected foals.

## Supplementary Material

Supplemental material is available online at www.g3journal.org/lookup/suppl/doi:10.1534/g3.117.039511/-/DC1.
