# Supplementary material for: A Nonsense Variant in the ST14 Gene in Akhal-Teke Horses with Naked Foal Syndrome
Source: G3 (Bethesda). 2017 Feb 22;7(4):1315–21. doi: 10.1534/g3.117.039511 (PMC5386879; doi:10.1534/g3.117.039511)
Supplement: Supplementary file 1 [file 1315FigureS1.pdf]

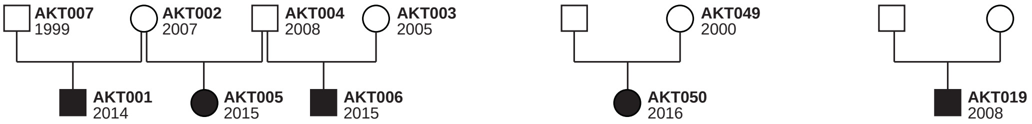

Figure S1 Pedigree of Akhal-Teke horses used for the study. Animals, from which samples were available, are indicated by laboratory identifier and birth year. We used a family consisting of 3 affected offspring and their 4 non-affected parents for linkage and homozygosity mapping (shown on the left). DNA from the affected foal AKT050 became only available after the linkage and homozygosity mapping were completed. From the affected horse AKT019, only an archived hoof was available. The DNA of this material was heavily degraded and proved to be insufficient for SNP chip genotyping. Therefore, this animal could also not be included in the homozygosity mapping. However, we confirmed in all five affected horses that these animals were homozygous for the *ST14:*c.388G>T variant.
